# Impact of Patient- and Surgeon-Related Factors on Weight Loss after Laparoscopic Sleeve Gastrectomy—A Single-Center Study

**DOI:** 10.3390/medicina60091450

**Published:** 2024-09-04

**Authors:** Mateusz Wityk, Natalia Dowgiałło-Gornowicz, Maciej Bobowicz

**Affiliations:** 1 Department of General and Oncological Surgery, Regional Health Centre, 59-300 Lubin, Poland; mateuszwityk@gmail.com; 2 Department of General, Minimally Invasive and Elderly Surgery, University of Warmia and Mazury in Olsztyn, Niepodleglosci 44 Str., 10-045 Olsztyn, Poland; 3 Department of Radiology, Medical University of Gdansk,17 Smoluchowskiego Str., 80-211 Gdansk, Poland; maciej.bobowicz@gumed.edu.pl

**Keywords:** laparoscopic sleeve gastrectomy, LSG, obesity surgery, surgeon-related factors

## Abstract

*Background and Objectives*: Surgical treatment for obesity is becoming increasingly popular. Surgeons have been trying to find a simple way to predict the type of surgical intervention that is best for a specific patient. This study aimed to determine the patient- and surgeon-related factors that affect weight loss after laparoscopic sleeve gastrectomy (LSG). *Materials and Methods*: A total of 129 patients underwent LSG in one surgical department. The following factors were analyzed: gender; age; highest preoperative and 6-month postoperative weight; the occurrence of obesity-related diseases, such as type 2 diabetes and hypertension; the number of surgeons involved in the surgery; and who performed the surgery, a resident or specialist. The outcomes also included length of hospital stay, operative time and complications. Statistical significance was defined as *p* ≤ 0.05. *Results*: A total of 129 patients (94 female) with a median age of 43 years and BMI of 43.1 kg/m^2^ underwent LSG, while a total of 109 (84.5%) patients achieved ≥50% of excess BMI loss (%EBMIL). Preoperative weight loss had no impact on %EBMIL (*p* = 0.95), operative time (*p* = 0.31) and length of hospital stay (*p* = 0.2). Two versus three surgeons in the operating team had no impact on surgery time (*p* = 0.1), length of stay (*p* = 0.98) and %EBMIL (*p* = 0.14). The operative time and length of hospital stay were similar for specialists and surgeons in training. %EBMIL was higher in the residents’ surgery without statistical significance (*p* = 0.19). Complications occurred in 3.9% without mortality or leaks. *Conclusions*: Preoperative comorbidities, surgeons’ experience and the number of surgeons in the operating team do not impact the complication rate, length of hospital stay, operative time and postoperative weight loss after LSG.

## 1. Introduction

Obesity has emerged as a global epidemic, giving rise to significant public health concerns due to its correlation with a multitude of chronic conditions, such as cardiovascular diseases, diabetes and specific cancers. According to the World Health Organization (WHO), the global prevalence of obesity has nearly tripled since 1975, with over 650 million adults classified as obese in 2016 [1]. This alarming trend is not limited to adults, as childhood obesity has also experienced a significant surge, with over 340 million children and adolescents aged 5–19 classified as overweight or obese. Global data show that the average life expectancy of patients suffering from obesity may be shortened by approximately 5–20 years [2,3,4].

The escalating prevalence of obesity is driven by a multifaceted interplay of factors, encompassing genetic predisposition, sedentary lifestyles, unhealthy dietary habits and environmental influences. The health ramifications of obesity are profound, resulting in a diminished quality of life and heightened healthcare costs [5,6]. In this context, bariatric surgery has garnered attention as an efficacious intervention for achieving substantial and enduring weight loss in people with obesity [7].

The number of bariatric and metabolic procedures performed worldwide is increasing every year. Surgical treatment for obesity is becoming increasingly popular due to its well-known and established positive effects on weight loss, obesity-related disease remission, improvements in quality of life and increased life expectancy [8,9,10]. New data also show a protective effect against particular types of cancer [8,9,10,11,12]. According to The International Federation for the Surgery of Obesity and Metabolic Disorders (IFSO), trends in the treatment of obesity have changed in recent years and, currently, laparoscopic sleeve gastrectomy (LSG) is the most frequently performed bariatric procedure [13,14]. Polish data also show the highest use of LSG among all types of obesity surgeries [15].

LSG can be performed by surgical teams with varying compositions, depending on the country, hospital and type of health insurance. While it is possible for the procedure to be carried out by a single surgeon using a single port, these are isolated reports. In practice, the surgery is most commonly performed by two or three surgeons. When two surgeons are involved, an automatic retractor may be used, or in some cases, no retractor is needed at all. In a team of three surgeons, one acts as the primary operator, the second as the assistant and the third holds the liver retractor. However, there are no precise statistical data on this topic [16,17].

In each medical field, there is an effort to identify factors that may impact the outcome of the medical intervention and translate them into a simple therapeutic algorithm. Bariatric and metabolic surgeons have been trying for many years to find a simple way to predict what type of surgical intervention will be best for a specific patient and what patient-related factors may influence ultimate weight loss, obesity-related disease remission or quality of life improvement [18,19,20,21].

This study aimed to identify the patient- and surgeon-related factors that influence weight loss outcomes following LSG in the short-term follow-up. Specifically, it sought to analyze how various demographic, clinical and technical factors contribute to the effectiveness of the procedure in terms of weight reduction. The secondary aims focused on the occurrence of postoperative complications. These complications were examined in relation to the same patient- and surgeon-related factors. By exploring these associations, this study aimed to provide insights into which factors might increase the risk of adverse events after LSG, thereby informing strategies to improve patient safety and surgical outcomes.

## 2. Materials and Methods

### 2.1. Patients

In this retrospective cohort study, 129 LSG were performed by the same team of surgeons between January 2019 and July 2020. Each patient who qualified for surgery met international criteria for surgical treatment of obesity [22]. The inclusion criteria were BMI over 40 kg/m^2^ or BMI over 35 kg/m^2^ with obesity-related diseases: type 2 diabetes (T2D), hypertension (HT), obstructive sleep apnea or hypercholesterolemia; another criterion was consent to participate in the study. The exclusion criteria were refusal to take part in the study and follow-up, or failure to meet the criteria for surgical treatment. Standard preparation for surgery in our center is 6 months. During this time, patients should lose weight and change dietary habits. There are no defined criteria for weight loss. Each patient is prepared by a multidisciplinary team consisting of a surgeon, internist, dietitian and psychologist. Data were collected preoperatively and during in-person visits six months after surgery, according to a standard postoperative protocol. The following patient factors were taken into account: gender, age, height, preoperative and postoperative weight and the occurrence of obesity-related diseases: T2D and HT. The outcomes were expressed as the percentage of excess BMI loss (%EBMIL). The surgeons’ factors were as follows: the number of surgeons involved in every surgery and who performed the surgery, a resident or specialist. If the operator was a resident, they were assisted by a bariatric surgery specialist. The outcomes also included length of hospital stay (LOS) expressed in days, operative time and complications according to the Clavien–Dindo scale. All outcomes were reported according to standard outcome criteria from the American Society for Metabolic and Bariatric Surgery (ASMBS) [23]. Remission of T2D is normal measures of glucose metabolism (HbA1c < 6%, fasting blood glucose (FBG) < 100 mg/dL) in the absence of antidiabetic medications. Remission of HT is being normotensive (blood pressure (BP) < 120/80) off antihypertensive medication.

### 2.2. Surgical Technique

Each surgery was performed using the same surgical instruments, including staples and high-energy devices (Signia™ Stapling System, LigaSure™, Medtronic, CT, USA; Maryland Jaw Laparoscopic Sealer/Divider, Medtronic, CO, USA). Pneumoperitoneum was created using an optical trocar inserted into the mid-abdomen. The pneumoperitoneum pressure was set at 12 mmHg in each case. Three 12 mm trocars and one or two 5 mm trocars were used for the operation. For hemostasis and dissection, the same tool utilizing bipolar energy was used in all operations. The gastric sleeve was calibrated using a 34F bougie, with a vertical gastric dissection starting 5 cm from the pylorus and finishing 1 cm lateral to the angle of His. In most cases, 5 staplers were used for gastric transection. In each case, the resected stomach was removed from the abdominal cavity through a 12 mm wound in the right mid-abdomen. The staple line was not reinforced with any suture, clip or glue. The methylene blue leak test was negative in all cases. To ensure proper hemostasis, after removal of the stomach specimen from the peritoneal cavity, blood pressure was pharmacologically raised by the anesthesiology team above 140/90 mmHg, and complete evacuation of the pneumoperitoneum was performed. After excluding bleeding from the abdominal tissues, the trocars were removed under visual control, and any bleeding was assessed. If three surgeons were involved in the operation, one was the primary surgeon, the second was the assistant and the third retracted the liver. If two surgeons participated in the surgery, no additional liver retraction was performed so there was one less trocar. Absorbable skin sutures were used to close the wounds, and no additional fascial sutures were used. A radiological leak test with oral contrast administration was not performed in any case.

All patients were managed in accordance with ERASB guidelines. Two hours before surgery, patients were given a high-carbohydrate oral drink. The surgeries were performed in the beach chair position. All patients received mechanical and pharmacological antithrombotic prophylaxis. Every patient qualified for early rehabilitation within the first hour after surgery. An oral liquid diet was initiated on the day of the procedure. The decision to discharge was made based on good pain tolerance, effective oral intake, proper mobilization and normal parameters such as blood pressure, heart rate and blood oxygen saturation. Laboratory blood tests were not performed as a standard procedure after surgery.

### 2.3. Statistical Analysis

All data were analyzed using Statistica software 13.PL (StatSoft Inc., Hamburg Germany). Descriptive statistics were used. The mean and standard deviations were estimated. For evaluating normality and equality of variances, the Shapiro–Wilk test and the Levene’s tests were used. The Mann–Whitney U test and the Kruskal–Wallis tests were used for the evaluation of the differences between variables. *p* values  ≤ 0.05 were considered statistically significant.

## 3. Results

A total of 129 patients (94 female, 72.7%) were analyzed. The mean age was 42.6 years and the mean preoperative BMI was 43.4 kg/m^2^, Table 1. Six months after surgery, 109 (84.5%) of 129 patients achieved 50.0% or greater %EBMIL, and the remaining 20 (15.5%) patients achieved %EBMIL of less than 50%.

### 3.1. Preoperative Weight Loss

The mean preoperative weight loss during the standard six-month preoperative workup was 9.9 kg ± 8.4 kg. In 22 patients, preoperative weight remained unchanged. Preoperative weight loss had no impact on %EBMIL (*p* = 0.95), length of surgery (*p* = 0.31) and LOS (*p* = 0.2).

### 3.2. Obesity-Related Diseases

**Figure 1:**
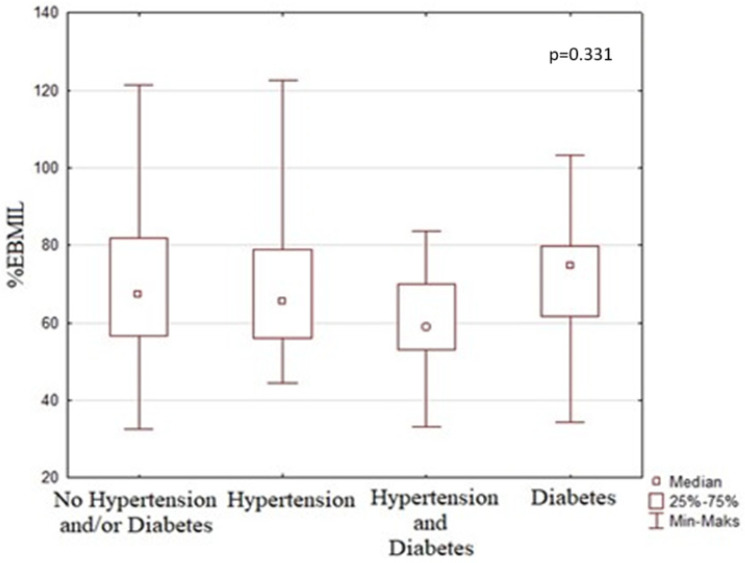
Comparison of preoperative comorbidities occurrence and postoperative %EBMIL.

While 43 (33.3%) patients had only HT and 9 (7.0%) patients had only TD2, 19 (14.7%) patients had both HT and T2D before the surgery. The presence of HT or T2D was associated with lower postoperative %EBMIL, especially in patients with both HT and T2D, but this was not statistically significant (Figure 1).

### 3.3. Age

Age was inversely related to %EBMIL (r(Spearman) = −0.26, *p* = 0.003). There was no correlation between age and operative time (r = 0.02, *p* = 0.78) or LOS (r = 0.02, *p* = 0.86).

### 3.4. Complications and LOS

Complications occurred in five patients (3.9%). There were three men and two women. Two of them had intraabdominal hemorrhage requiring reoperation on the day of primary surgery–Clavien–Dindo grade IIIb. In both cases, the exact source of bleeding was not located. There was no active bleeding intraoperatively. Peritoneal lavage and drainage were performed. No further bleeding was observed postoperatively. The hemoglobin level in the control blood laboratory tests remained stable. The oral diet was reintroduced with good tolerance on the first postoperative day. Further hospitalization was uneventful in both cases, and the overall LOS was six days. None of the patients required a blood transfusion. One patient had a superficial wound infection which was successfully treated at an outpatient clinic–Clavien–Dindo grade I. The wound of the right mid-abdomen became infected at the place where the stomach specimen was removed from the peritoneal cavity. The wound was opened and cleaned under aseptic conditions. After proper healing was achieved on the 7th day, the wound was closed with delayed sutures. No systemic antibiotic therapy was used. No further wound-related complications were observed. During the wound treatment period, the patient’s condition remained stable. Two patients required rehospitalization due to gastrointestinal infection and abdominal pain. Two days after discharge, the patients experienced symptoms of vomiting, fever, diarrhea and general weakness. After admission to the ward, anti-peristaltic, anti-emetic treatment and intravenous hydration were introduced, resulting in a quick improvement in the general condition and disappearance of symptoms. Both cases were not related directly to surgery and were classified as Clavien–Dindo grade I. Among the complication group, two patients had HT, and one patient had T2D. All complications occurred after surgeries performed by specialists. There was no mortality or leak.

The limited rate of data compilation prohibits a robust statistical analysis and the identification of significant correlations between the occurrence of complications and factors associated with the patient or surgeon.

### 3.5. Surgeon Factors

All surgeries were performed by teams of two or three surgeons. The number of surgeons in the operating team had no impact on operative time (*p* = 0.10) or LOS (*p* = 0.98). %EBMIL was slightly higher in patients with LSG performed by three surgeons, but it was not statistically significant (*p* = 0.14) (Table 2).

A total of 29 (22.5%) LSG were performed by surgeons in training. Preoperative BMI was significantly lower in patients operated on by residents (*p* = 0.009), with no statistical difference in obesity-related diseases. Operative time and LOS were similar for both specialists and surgeons in training (*p* = 0.05 and *p* = 0.87). %EBMIL tended to be higher in patients operated on by residents, but this was not statistically significant (*p* = 0.19), Table 2.

## 4. Discussion

Our study indicated that LSG (laparoscopic sleeve gastrectomy) can be a highly effective weight loss procedure with a low complication and rehospitalization rate, even in the early stages of follow-up. In specific cases involving patients with a lower BMI, surgery performed by a surgeon in training may be as safe and effective as that performed by a specialist. Our findings also suggest that there is no significant difference between surgeries performed by a team of three surgeons and those performed by two surgeons.

In our study, we use %EBMIL to express the outcomes. This was calculated with the following formula:

$\frac{preoperative\;BMI-actual\;BMI}{preoperative\;BMI-25}\times 100$ [23]. This measures the proportion of excess BMI lost relative to a patient’s initial weight, providing a standardized way to assess weight loss outcomes across different populations and surgical procedures. %EBMIL is a commonly reported outcome in different studies [24,25,26,27,28].

Our observations are consistent with previously published data. Fuks et al. achieved a 38.6% reduction in excessive body weight loss 6 months after surgery, with a complication rate of 5.1% in 135 patients [29]. Kelaidari et al. showed a 69.2% excess weight loss (%EWL) in 35 patients with three cases of gastric fistula [30]. Mean %EBMIL of 65.24% and reduction in metabolic syndrome prevalence in 55.9% of cases in 124 patients were described by Sirbu et al. [24]. The positive effect of younger age on %EBMIL after bariatric surgery in early follow-up was described by other authors [25,26,27,28]. However, Gonzalez-Heredia et al. showed no significant correlation between age and weight loss [31].

Some authors have attempted to investigate perioperative factors associated with good weight loss outcomes after bariatric surgery [21,32,33,34,35,36,37]. Cottam et al. found that preoperative BMI, age, hypertension and diabetes impact weight loss with a significant impact of HT and T2D coexistence, which is partially comparable to our observations [32]. Chun-Wei Huang et al. showed that younger age was associated with better outcomes [36]. In their study, other positive predictors of weight loss were no alcohol consumption and no mental health disorders. Woźniewska et al. found that age had no impact on 2-year weight loss after LSG, but being under 45 was associated with more rapid improvement in carbohydrate and lipid profiles [38]. Gomberawalla et al. described patients with a BMI below 50 kg/m^2^ as having the greatest chance of weight loss success with LSG [39].

According to data from 22,327 patients from the Scandinavian Obesity Registry, preoperative weight loss was associated with greater postoperative weight loss and a lower risk of complications [40]. However, this observation could not be confirmed in the analysis of 349 016 bariatric patients conducted by Tewksbury et al. The authors also concluded that unsafe weight loss before surgery may be associated with higher rates of infections [41].

The overall complication rate in this study was low (3.9%), with no significant difference in outcomes between surgeries performed by specialists versus those performed by residents in training. Notably, all complications occurred in surgeries conducted by specialists, but the small sample size and low complication rate make it difficult to draw definitive conclusions about the relationship between surgeon experience and complication rates. Major et al. and Goldberg et al. tried to assess the impact of surgery performed by trainees on complication rate, operative time and weight loss outcomes [42,43]. In opposition to our study, Goldberg showed that surgeon’s experience had a significant impact on LSG outcomes [42]. The authors found higher rates of readmission, reintervention and complication with more frequent leaks after surgeries performed by residents [42]. Major et al. found no correlation between surgery performed by trainees and the frequency of complications and reoperations [43]. Weight loss outcomes were similar in both groups. Contrary to our results, the operating time was significantly longer in the resident group, but comparisons of this variable are not possible due to the lack of stratification of surgeons’ experience in the training groups [43].

The strength of this study is that it is one of the first to evaluate the impact of the number of surgeons in the surgical team on the outcomes of LSG surgery. Therefore, it is not possible to compare it with published evidence. In the authors’ opinion, the assessment of technical aspects of surgery, such as the composition of the surgical team and its possible impact on the occurrence of complications, helps to obtain better surgical results and reduce the overall costs of care in the surgical treatment of obesity. It is an important aspect of perioperative care and may influence decision-making in the healthcare system.

This study’s main limitations are the small number of patients and the retrospective nature of the analysis, which may limit the generalizability of the findings. This is usually an issue for new centers, but we believe that performance auditing and research are very important for both the surgical team and our patients because it impacts the quality of care. Despite these limitations, this study provides valuable insights, particularly regarding the safety and efficacy of surgeries performed by residents and the minimal impact of surgeon team size on outcomes.

## 5. Conclusions

LSG is a good surgical option for obesity treatment according to short postoperative observation. The procedure had a low complication rate with no mortality. No significant differences in outcomes were found between surgeries performed by specialists and residents. Preoperative weight loss, occurrence of HT and T2D, surgeon experience and the number of surgeons in the operating team did not impact the complication rate, LOS, operative time or postoperative weight loss. Further observation and analysis of data regarding longer postoperative follow-up are necessary to verify and confirm the conclusions.

## Figures and Tables

**Table 1:** Characteristics of patients (SD standard deviation; BMI body mass index).

Variable	Value
Female/Male, *n* (%)	94/35 (72.9%)
Age [years] ± SD (range)	42.6 ± 11.1 (22–69)
Preoperative BMI [kg/m^2^] ± SD (range)	43.4 ± 5.3 (33.9–66)
Preoperative weight [kg] ± SD (range)	123.2 ± 19.4 (88–186)
Preoperative weight loss [%] ± SD (range)	7.2 ± 5.8 (0–27.9)
Operative time [min] ± SD (range)	69.7 ± 22.1 (35–145)
Length of hospital stay [days] ± SD (range)	3.1 ± 0.4 (2–6)

**Table 2:** Specialists’ vs. surgeons’ training outcome comparisons (%EBMIL percentage of excess body mass index loss).

Variable	Surgeon	Value (Min–Max)	*p* Value
Preoperative weight	Specialist	124.50 (89.0–186.0)	0.009
	Resident	112.0 (88.0–165.0)	
%EBMIL	Specialist	65.1 (32.8–122.7)	0.19
	Resident	69.5 (47.0–104.1)	

## Data Availability

The original contributions presented in the study are included in the article; further inquiries can be directed to the corresponding author/s.
